# Gestational Diabetes and Obesity: Immediate and Late Sequelae for Offspring

**DOI:** 10.3390/children12091263

**Published:** 2025-09-19

**Authors:** Maria Kaza, George Paltoglou, Kalliopi Rodolaki, Konstantinos Kakleas, Spyridon Karanasios, Kyriaki Karavanaki

**Affiliations:** 1 Diabetes and Metabolism Unit, Second Department of Pediatrics, National and Kapodistrian University of Athens, “P&A Kyriakou” Children’s Hospital, 115 27 Athens, Greece; mka274274@gmail.com (M.K.);; 2 First Department of Pediatrics, National and Kapodistrian University of Athens, “Aghia Sophia” Children’s Hospital, 115 27 Athens, Greece

**Keywords:** pregnancy, obesity, macrosomia, hyperglycemia, gestational diabetes mellitus (GDM)

## Abstract

**Highlights:**

**What are the main findings?**

- Gestational diabetes and obesity are followed by an interplacental variety of complications responsible for maternal morbidity and fetal reprogramming.
- Although gestational diabetes is an area of scientific research in the past decade mainly due to short- and long-term maternal and fetal implications, there is a lack of knowledge in regard to therapeutic alternatives such as antioxidant supplementation and its long-term effectiveness.

**What is the implication of the main finding?**

- We recommend antioxidant supplementation during preconception and gestation for mothers with pregestational diabetes or a previous history of gestational diabetes.
- The use of continuous glucose monitoring systems, dietary modifications during pregnancy and regular mild–moderate exercise against oxidative stress during gestation and as a preconception modification are valuable assets against gestational diabetes- and diabesity (gestational diabetes and obesity)-related complications.

**Abstract:**

**Background/Objectives**: Gestational diabetes mellitus (GDM) and maternal obesity are major global health gestation-related conditions associated with several adverse maternal and neonatal outcomes. GDM is a common gestational metabolic disorder, presented usually during the second or third trimester of pregnancy with maternal hyperglycemia due to insulin intolerance. Maternal obesity, defined as a BMI of a woman during gestation ≥ 30 kg/m^2^, has been associated with maternal complications such as GDM, fetal macrosomia and others. **Methods:** The presented article is a narrative review. The aim of this study was to review scientific evidence and conduct a comprehensive analysis of GDM and maternal obesity (“diabesity”) and its immediate and late complications for both maternal and fetal/offspring wellbeing. **Results:** This review highlighted that gestational hyperglycemia results in oxidative and nitrogen stress development and that maternal obesity may have an impact similar to maternal diabetes, as it may cause fetal macrosomia and cardiometabolic complications later in life. **Conclusions:** Optimal diabetic control is responsible for the prevention of oxidative stress in diabetic pregnancies. Similarly, pregnant women should exercise regularly, receive folic acid supplementation and avoid excess weight gain during pregnancy. Breastfeeding during the first months of life has a positive impact on weight monitoring in infants born to mothers with diabesity and may be crucial in the prevention of obesity and metabolic syndrome later in life.

## 1. Introduction

The presence of diabetes during gestation is characterized by the disordered secretion and metabolism of insulin, leading to hyperglycemia. As expected, the aforesaid disorder implicates the overall maternal, fetal and neonatal health, posing a significant threat and resulting in grave morbidity. A previously normoglycemic woman may be first diagnosed with diabetes during the gestational period. Gestational diabetes mellitus (GDM) is defined as any type of glucose intolerance during pregnancy, especially during the second and third trimesters of pregnancy compared to other types of diabetes, existing either prior to pregnancy (PGDM) or in the early stages of pregnancy (usually the first trimester). As a result, the diagnostic approach of diabetes should include other types, such as type 1 diabetes mellitus (T1D), late autoimmune diabetes mellitus in adults (LADA), type 2 diabetes (T2D), maturity onset diabetes of the young (MODY), etc. [1,2,3,4].

As the American College of Obstetricians and Gynecologists markedly states, GDM is one of the most common medical complications of pregnancy [5]. Moreover, the global prevalence of GDM is estimated to be 14% [95%CI (confidence interval): 13.97–14.04%] [6]. Interestingly, high-income countries exhibit the highest standardized prevalence of the disease [6]. GDM is best described as a glucose intolerance detected at any time during pregnancy, responsible for serious maternal and fetal complications. The pathophysiological mechanism of the disorder includes the development of maternal hyperglycemia due to inadequate insulin secretion, typically accompanied by insulin resistance during the second or third trimester of pregnancy [7,8,9]. In many instances, hyperglycemia results from impaired glucose tolerance due to pancreatic β-cell dysfunction on a background of chronic insulin resistance. As pregnancy progresses, insulin resistance is promoted by a surge of local and placental hormones, including estrogen, progesterone, leptin, cortisol, placental lactogen and placental growth hormone [7,8,9]. Risk factors for GDM include overweight and obesity, advanced maternal age, Westernized diet, micronutrient deficiencies and a family history or any form of diabetes [10].

Although GDM is usually resolved post-labor, this condition may lead to severe health complications later in life, such as increased risk for type 2 diabetes (T2D) and cardiovascular disease (CVD) for the mother and future development of obesity, CVD, T2DM and/or GDM for offspring, which is carried to future generations through epigenetics. This results in a vicious cycle of obesity and diabetes, which contributes to the increase in obesity and diabetes pandemics [2].

According to the American Diabetes Association (ADA) 10% of pregnancies are affected by GDM per year, whereas the estimated prevalence of GDM is 10–14% (World Health Organization) [11,12]. Moreover, as previously highlighted, according to the International Diabetes Federation (IDF) estimates, GDM affects approximately 14% of pregnancies worldwide, representing approximately 18 million births annually, and this number is set to rise along with the escalating obesity epidemic [6,13]. Thus, GDM, as a major global health gestational disorder, poses a great obstetric challenge that needs to be seriously addressed by the global health community due to the immediate and long-term maternal- and offspring-related complications and its major social and economic impact in communities. Therefore, studies highlight the importance of adopting a healthy lifestyle as a preventive measure against the development as well as management of GDM [10,14]. Thus said, a healthy lifestyle includes weight surveillance, healthy diet and regular exercise as a preventive measure of the development of GDM and a key regulator in management of diabetes during pregnancy [10,14,15,16].

From another perspective, maternal obesity has become an increasingly prevalent issue for the obstetric community against the medical recommendations given by obstetricians, advocating for healthy eating and weight management for women during gestation and the preconceptional period. According to the World Health Organization (WHO) definition of obesity (2018), it is a situation that includes the excessive accumulation of fat in adipose tissue that may potentially cause health impairment [17].

Obesity exhibits a rapidly increasing prevalence worldwide and can be caused either by genetic factors (severe-type monogenic and genetic syndromic obesity) or can be multifactorial, flourishing in obesogenic environments. Although Asian populations require a lower BMI cut-off point, the WHO defines overweight as a Body Mass Index (BMI) of 25–29.9 kg/m^2^ and obesity as a BMI ≥ 30 kg/m^2^ [17]. Maternal obesity may cause serious complications for both the mother and the fetus. Those include higher rates of maternal morbidity, GDM, hypertensive disorder and fetal congenital anomalies [18,19,20]. Consequently, maternal obesity has a heavy socioeconomic impact, which includes immediate- and late-obesity-related maternal and fetal sequelae [18,19,20,21].

Although GDM and maternal obesity are undoubtedly a broad field of increasing scientific interest over the past few decades, there is a clear need for updated review studies that incorporate the newly obtained data in the GDM and obesity research field while embracing and evaluating the results of previous studies. Moreover, the ongoing research on the consequences of gestational obesity underlines the necessity for weight management during pregnancy. To our knowledge, this review contributes significantly to the existing literature by incorporating newer studies into the previous knowledge we have on obesity and diabetes during gestation and their maternal and fetal consequences as a result of hyperinsulinemia, oxidative stress and fetal reprograming. In addition, this review takes a step further and explores the preventive measures and modifiable factors of diabesity (maternal obesity and gestational diabetes). Hence, this review presents a comprehensive presentation of the complications (immediate and late) for the mother and the child yet also introduces all the latest recommendations for the prevention of GDM and the treatment of oxidative stress with nutritional interventions and supplements.

## 2. Materials and Methods

The presented article is a narrative review. Through this narrative review, we studied the factors associated with maternal obesity and GDM and discussed the potential underlying mechanisms of the observed associations and the immediate and late complications for the mother and the offspring. The aim of this study was to review the late scientific evidence along with the previous data in order to conduct an up-to-date comprehensive analysis of GDM, maternal obesity and its immediate and late maternal and fetal/offspring complications. This review has been conceptualized as the need for an up-to-date combined review of the mechanisms, complications and prevention of GDM and maternal obesity has emerged, aiming to include all the new data in a simple and comprehensive manner.

A literature search in the electronic databases PubMed, Scopus and Google Scholar was performed by two researchers (M.K. and G.P.), independently. An analysis of eligible publications in the literature using MESH terms such as “gestational diabetes mellitus”, “GDM complications”, “maternal obesity” and “gestational hyperglycemia” as keywords was conducted.

The aforementioned research emphasized more recent scientific data (2015–2025) and combined previous data with more recent data, aiming to present the latest scientific results. The references included in these selected publications were also considered to find additional relevant articles. Inclusion criteria were as follows: a. articles in English; b. publication date 2015–2025; c. population-based studies, reviews, systematic reviews, meta-analyses and clinical trials. Scientific works that were not presented in English and/or were not relevant were excluded from our review.

Thus said, the search for gestational diabetes included at least 7767 results and maternal obesity 7419 results (July 2025). Duplicates and pertinence were deemed according to title and abstract if available. Full-text articles for all relevant studies were reviewed. Following independent revision, the researchers (M.K. and G.P.) met to resolve disagreement by discussion. In the absence of consensus, a third researcher reviewed the article (K.K.).

## 3. Results

The literature search was performed in three stages.

A. Initially, articles on GDM prevalence, pathogenesis, oxidative stress and its associations with acute and chronic complications for the mother and offspring were searched in the databases. Of the initially found 90 articles, 5 were duplicates and 12 were excluded by title and abstract, and thus 73 remained.

B. Secondly, articles on obesity in pregnancy and its effects on the mother and offspring were searched in the databases. Of the initially found 23 articles, 2 were excluded by abstract and title and 21 remained.

C. Finally, articles on GDM management and prevention strategies were searched on in the databases. Of the initially found 35 articles, 5 were excluded by abstract and title and 5 were duplicates; thus, 25 remained. In total, 119 articles were included in the references. After consideration, all three researchers agreed on the use of a total of the above 119 articles (Table 1. Prisma Flow diagram).

### 3.1. Gestational Diabetes Mellitus (GDM)

#### 3.1.1. GDM Demographics

As previously highlighted, GDM poses a great obstetric challenge worldwide, as according to the ADA 10% of pregnancies are complicated annually by GDM [11,12]. Moreover, the IDF estimates that as many as 14% of pregnancies are impacted by GDM worldwide [6,13].

Therefore, GDM represents the most common type of diabetes during pregnancy, occurring in up to 1.7–28% of pregnancies (depending on population and diagnostic criteria) [22,23,24,25]. The constant rise in GDM prevalence worldwide can be attributed to increasing maternal age and obesity rates, as well as sex and nationality differences, and has serious social, economic and personal consequences for both the mother and her offspring [22,23,24,25].

#### 3.1.2. GDM Diagnosis

The diagnosis of GDM is based on the HAPO (Hyperglycemia and Adverse Pregnancy Outcome) study recommendations, which are mentioned in Table 2 [26]. Following the confirmation of pregnancy and as early as the first prenatal appointment to the maternal clinic, a fasting glucose measurement (following 8 h of fast) is performed. Blood glucose levels > 126 mg/dL are indicative of pregestational diabetes mellitus. Blood glucose levels between 92 and 125 mg/dL are considered diagnostic of GDM. Blood glucose levels < 92 mg/dL require further testing with the 75gr glucose oral tolerance test (75 OGTT), performed at 24–28 gestational weeks (Table 2).

#### 3.1.3. Predisposing Modifiable Factors Related to GDM

The predisposing factors associated with the development of GDM include unmodifiable and modifiable elements. The modifiable predisposing factors are potentially preventable and therefore form an extremely important category that should be properly addressed by the clinician. These factors include excessive weight gain/obesity, unhealthy rich in fat diet, hypertension, endocrine disorders such as polycystic ovaries syndrome (PCOS) and sedentary lifestyle.

As all of the aforementioned factors can be reformed, it is evident that GDM may be prevented with lifestyle modifications (weight loss and/or reduced weight gain during pregnancy, healthy diet and exercise) and by addressing any possible endocrinological disorders, such as preconceptual weight management and metformin therapy in women with PCOS [27].

#### 3.1.4. Predisposing Unmodifiable Factors Related to GDM

The unmodifiable factors that predispose in GDM include a growing area of scientific research. This category includes factors that cannot be changed or prevented with active intervention by a clinician. These factors include genetic predisposition, progressed maternal age (pregnancies over 35 years old), prior history of GDM in pregnancy and race [28,29].

**A. GDM-RELATED MATERNAL COMPLICATIONS**

GDM may be associated with serious maternal- and offspring-related complications. Research data highlight that GDM is a strong predictor of these health issues compared to non-GDM individuals [30,31]. Maternal complications may be present during the gestational period, post- and/or during labor or as long-term complications and are listed in Table 3(a).

GDM-related gestational complications for the mother include gestational stress, pre-eclampsia, hypertensive and circulation disorders, infections and sepsis. Post-labor complications include shoulder dystocia or caesarean section due to fetal macrosomia, premature delivery and increased risk of maternal mortality.

Individuals with gestational diabetes mellitus (GDM) appear to be at significantly increased risk of developing GDM in future pregnancies, T2D in the next decade and cardiovascular disease later in life compared to healthy populations. Interestingly, the long-term complications include a 7-fold increased risk of developing T2D in the next 10 years and increased risk of cardiovascular disease compared to the general population [32,33].

**Table 3:** (a) Maternal GDM-related complications (immediate and late sequelae) [34]. (b) Offspring GDM-related complications [after birth and later in life] [35].

**(a)**	
Immediate Consequences for the Mother	
Increased levels of oxidative stress during gestation Dystocia and/or caesarian section due to neonatal macrosomia Preeclampsia, hypertension and circulatory disorders during gestation Sepsis/perinatal infections Increased maternal morbidity/mortality	
Late Consequences for the Mother	
Increased risk of T2D Increased risk of metabolic syndrome Increased risk of cardiovascular disease	
**(b)**	
Fetal, Neonatal, Offspring, Adult Complications	
Fetal complications	Congenital anomalies, fetal death, macrosomia, cardiomyopathy, chorioamnitis, perinatal asphyxia, hydramnios (cause for preterm labor) and large for gestational age
Neonatal complications	Traumatic labor, respiratory difficulty, transient tachypnoea, hypoglycemia, polycythemia, jaundice, hypocalcemia, hypomagnesaemia and neonatal death
Pediatric complications	Neurodevelopmental complications, obesity, insulin resistance, impaired glucose tolerance, T2D and early onset of puberty
Adult complications	Obesity, insulin resistance, impaired glucose tolerance, type 2 diabetes, metabolic syndrome and depression

**B. PREGESTATIONAL DIABETES MELLITUS (PGDM) COMPLICATIONS FOR OFFSPRING**

Even though the risk of congenital complications caused by maternal diabetes can be eradicated by close glycemic surveillance, optimized glycemic control and prenatal monitoring, a great number of diabetic pregnancies eventually turn out to be unplanned.

Inadequately controlled (HbA1c > 7%) PGDM (usually T1D, T2D or MODY) can result in serious congenital defects (5–10% of pregnancies) and spontaneous abortions (15–20% of pregnancies) [36,37,38]. Interestingly, spontaneous abortions and stillbirths have also been correlated with higher BMIs and a higher risk of prediabetes [39,40,41].

Congenital malformations encountered in sub-optimally controlled PGDM involve serious cardiac defects (i.e., transposition of the great arteries, ventricular septal defects etc.), skeletal defects (hemivertebrae or caudal regression syndrome), neurological defects (anencephaly, meningocele syndrome, hydrocephalus, etc.), gastrointestinal tract defects (i.e., small left colon syndrome and situs inversus) and renal defects, like renal agenesis (Table 4). Results from observational studies underline that PGDM may also be associated with serious neurodevelopmental disorders, such as attention deficit-hyperactivity disorder (ADHD) and autism, fine and gross motor disorders and psychiatric diseases, including schizophrenia and anxiety disorder, manifested in childhood and adulthood [42]. Exposure to intrauterine hyperglycemia and increased oxidative stress holds significant risk of developing neurodevelopmental disorders during childhood as well as later in life. Thus said, inadequate glycemic control, especially during the first 10 gestational weeks (often associated with PGDM), when organ formation occurs, has been associated with neural-tube- and brain-formation-related disorders [43,44,45]. It is therefore wise for women with PGDM to schedule the timing of their pregnancy following the achievement of optimized glycemic control (HbA1c 7%) at least three months prior to conception [39,40].

**C. CONSEQUENCES OF UNCONTROLLED GDM ON THE FETUS, NEONATE AND CHILD**

GDM usually presents later in gestation and is often of milder severity compared to PGDM, primarily because GDM-related hyperglycemia develops during the second or third trimester of pregnancy. Nevertheless, the consequences of GDM are numerous, potentially leading to severe morbidity and mortality of the affected offspring and maternal morbidity (Table 3a,b). However, they are less in number and severity compared to PGDM, as GDM occurs after the first trimester of pregnancy, when organogenesis is already completed.

**D. CONSEQUENCES OF UNCONTROLLED GDM ON THE FETUS**

The fetal consequences of GDM are included in Table 3b. These complications are potentially very serious and can even threaten the life of the developing fetus, as they include fetal death and labor-related perinatal asphyxia.

Furthermore, GDM-related fetal complications include congenital anomalies, macrosomia, cardiomyopathy, chorioamnitis, hydramnios and large for gestational age GDM-related complications [after birth and later in life] [35].

**D1. Oxidative-stress-related mechanisms and GDM-related complications**

GDM-related complications mainly include nitride and oxidative stress pathways, relating to an imbalance between pro-oxidative and antioxidant cellular systems [48]. Diabetes-related oxidative stress mechanisms are responsible for the development of various complications, affecting the mother, the placenta, the fetus and the intrauterine fetal programming responsible for late and long-term metabolic disorders. Eventually, these lead to massive cellular destruction via disruption in protein and lipid metabolism and cellular DNA. Maternal hyperglycemia is responsible for the production of a high concentration of reactive oxygen species (ROS) and suppressed antioxidant fetal cellular protective mechanisms. These changes lead to premature placental aging, disordered morphology of endoplasmic reticulum and the formation of meningomyelocele. Such serious complications are more often encountered in PGDM neonates. Additionally, disordered mitochondrial activity leads to the formation of increased levels of ROS, and disrupted fetal-cell programming triggers apoptotic mechanisms, embryopathy and fetal death [49,50].

While oxidative stress holds a key role in maternal–fetal circulation, antioxidant mechanisms are essential in mitigating oxidative damage caused by various maternal conditions. Unfortunately, immature fetal antioxidant mechanisms facilitate fetal exposure to the harmful effects of oxidative stress, while the counteracting antioxidant mechanisms are obstructed as well, diminishing the formation of antioxidant enzymes. As a result, the function of platelets and cellular membranes may be seriously affected, resulting in damage in the maternal–fetal circulation [51,52].

Antioxidant substances protect the human body from the harmful effects of oxidative stress and can be divided into two groups (hydrophilic and hydrophobic). Hydrophilic substances include vitamin C, glutathione, lipoic acid and uric acid. Hydrophobic substances protect the membranes from hyper-oxidation and include carotenoids, vitamin E and coenzyme Q. The source of these antioxidant substances may be external, from a healthy diet, such as fruits and vegetables (rich source of vitamin C), or internal, where they are produced at the cellular level, such as enzymatic antioxidants. Enzymatic antioxidants reduce the toxic effects of H_2_O_2_ and enhance the formation of H_2_O [53,54,55,56,57].

GDM has been held responsible for a long list of related complications. Among these, the most important and well-studied are listed below and include cardiovascular and neurodevelopmental complications, as well as complications related to intrauterine growth retardation (IUGR) and macrosomia.

**D2. Macrosomia, intrauterine growth restriction and related complications**

Macrosomia is a neonatal diabetes complication defined as birth weight ≥ 4 kg (>97th percentile). Neonatal microsomia is defined as birth weight <2.5 at 40 weeks of gestation (<10th percentile). Both macrosomia and microsomia may occur in association with GDM, though macrosomia is far more common [58,59]. The underlying mechanisms include increased fetal adiposity and hyperinsulinemia as a consequence of increased circulating levels of glycose in diabetic pregnancies [58,59]. Therefore, there seems to be a strong correlation between elevated maternal glucose levels with high birth weight and elevated cord blood c-peptide levels.

Intrauterine growth retardation in uncontrolled diabetes (usually PGDM)-related pregnancies is due to severe placental vascular damage and diminished fetal–placental nutritional transfer, resulting in preterm delivery and other neonatal and long-term complications [60].

**E. CONSEQUENCES OF UNCONTROLLED GDM ON THE NEONATE**

For neonates born to mothers with poorly controlled GDM, there is a high risk for various perinatal complications and increased neonatal morbidity. These complications typically include prematurity, neonatal macrosomia or microsomia, respiratory distress syndrome, cardiomyopathy and others. The main cause of the above complications is increased oxidative stress, hyperproduction of ROS and hyperglycemia-related hyperinsulinemia.

The pathophysiological mechanism of neonatal macrosomia usually involves maternal hyperglycemia and subsequent neonatal hyperinsulinemia and is the main cause of perinatal asphyxia and meconium aspiration, polycythemia, hypoglycemia, hypocalcemia, hypomagnesemia and eventually neonatal death [61,62,63,64,65,66,67].

Neonatal hypoglycemia is the most frequent complication of GDM neonates, presenting during the first 24 h of life, and is associated with neonatal macrosomia (>90th centile) and hyperinsulinemia. The management of neonatal hypoglycemia includes periodic glucose level monitoring, neonatal rooming-in and frequent breastfeeding [41,42]. Neonatal hypocalcemia, occurring in 30% of GDM neonates, is associated with poor maternal glycemic control and impaired Ca, P and vitamin D metabolism, occurring mainly during the third trimester of pregnancy [61,62].

Neonatal polycythemia develops due to higher glucose uptake and high levels of erythropoietin [61,62,68,69]. Subsequently, the aforementioned changes lead to the development of secondary fetal hypoxia and hyperbilirubinemia due to increased fetal red cell production [61,62,68]. The complications of polycythemia occur due to excessive blood clotting and include feeding difficulties, plethora, cyanosis, lethargy, hypotonia, irritability, RDS, necrotizing enterocolitis, hyperbilirubinemia, hypoglycemia, seizure, thrombosis of renal and other arteries, kernicterus and encephalopathy [61,70,71].

Furthermore, neonates born to mothers with diabetes have an increased risk of developing neonatal respiratory distress syndrome due to reduced production of surfactant because of hyperinsulinism, transient tachypnea due to cesarian section and elevated risk for meconium aspiration and perinatal asphyxia due to macrosomia [63,64,65,66,67].

#### 3.1.5. Cardiovascular Complications

GDM has been related to an increased incidence of incipient hypertrophic cardiomyopathy. Usually, there is hypertrophy of the intraventricular septum, which occurs due to the toxic effect of oxidative stress caused by hyperglycemia, which usually subsides during the first 6 months of life. It is important to note that proper diabetic control does not exclude the possibility of cardiovascular complications in infants exposed to diabetes during pregnancy. The study of Mitanchez D et al. in 2014 included 80 women, of which 43 had diabetes and 37 were healthy and were evaluated at 24–28 gestational weeks [58,72]. According to the results, it was observed that the level of severity of oxidative stress was proportional to cytokine levels (↑TNF-α and ↑Il-10) and to the reduction in left ventricular contractility (*p* = 0.001). The researchers concluded that all infants exposed to either gestational or pregestational diabetes should have frequent assessment of their cardiac function [58,72].

**F. LONG-TERM CONSEQUENCES OF UNCONTROLLED GDM ON THE CHILD AND ADULT**

The long-term GDM-related sequalae on offspring include psychiatric and neurodevelopmental disorders, cardiovascular complications, metabolic syndrome and obesity [42,73,74,75].

**F1. Neurodevelopmental and psychiatric disorders**

Moreover, as shown in Table 3a,b, children with a history of intrauterine exposure to GDM may present a heightened risk of certain neurodevelopmental disorders, potentially caused by diminished neurotrophin levels responsible for the development and maturation of the fetal–placental unit and fetal growth [76,77,78]. These neurodevelopmental disorders include autism, ADHD, reduced cognitive function and psychiatric diseases [42,73,74,75]. The Danish National Birth Cohort study included 2,413,000 births between 1978 and 2016. The results retrieved from this study showed that 151,200 participants suffered from mental illnesses, from which 52,206 of them had a history of exposure to diabetes in the gestational period [74]. It was noted that offsprings of diabetic mothers had increased risk of mental disorders (HR = 1.15) and, more specifically, schizophrenia (HR = 1.55), anxiety disorders (HR = 1.22), cognitive disorders (HR: 1.29), neurodevelopmental disorders (HR:1.16) (ADHD, autism) and personality disorders (HR = 1.17). The results of this study were indicative of the association between intrauterine exposure to hyperglycemia and a higher risk of neurodevelopmental and psychiatric diseases for the next 40 years [74].

Exposure to intrauterine hyperglycemia and increased oxidative stress holds significant risk of developing neurodevelopmental disorders during childhood as well as later in life. Neurotrophins are a family of growth factors associated with the development and regulation of neurons, the development and maturation of the fetal–placental unit and fetal growth. Interestingly, GDM-related neurodevelopmental disorders have been associated with diminished neurotrophin levels [76,77,78]. As a matter of fact, Briana et al.’s study highlighted that brain-derived neurotrophic factor (BDNF) is down-regulated in fetuses exposed to GDM. Additionally, maternal diabetes is associated with nerve growth factor (NGF) deficiency in IUGR fetuses, which may be related to neurodevelopmental complications [76]. Furthermore, according to Jadhav et al.’s study, alterations in the levels of NGF and BDNF in the placenta of women with GDM may result in increased risk for late neurodevelopmental disorders for offspring [76]. However, these findings are based on observational studies and should be examined with caution.

The physiological function of neurotrophins promotes antioxidant mechanisms that support the proper development and functioning of the fetal nervous system. Nonetheless, maternal nutrition holds a key role in fetal growth and birth outcome. Therefore, the administration of Ω3 fatty acids and folic acid may prevent fetal neurological damage [74,75,76].

**F2. Obesity, Type 2 diabetes, metabolic syndrome and increased cardiovascular risk in adulthood**

In addition, neonatal macrosomia caused by gestational diabetes or maternal obesity may lead to the development of obesity, type 2 diabetes or metabolic syndrome later in adult life. GDM and obesity are responsible for the development of a chronic inflammatory state that involves the hyperproduction of proinflammatory cytokines and leptin, while there is reduced production of adiponectin [27,79,80]. Fetuses are therefore exposed to inflammatory cytokines, hyperglycemia, oxidative stress, exaggerated transfer of lipids through the placenta and reduced telomere length, which leads to increased risk for obesity and T2D in adults [27,75,80,81]. Moreover, there is the activation of the hypothalamic–pituitary–adrenal axis and subsequent elevation of angiotensin II levels and exaggerated oxidative stress. This in turn leads to enhanced production of reactive oxygen species, inhibition of vasodilators and activation of vasoconstrictors, all of which induce endothelial dysfunction and possibly hypertension in adult life [27,81].

**Figure 1:**
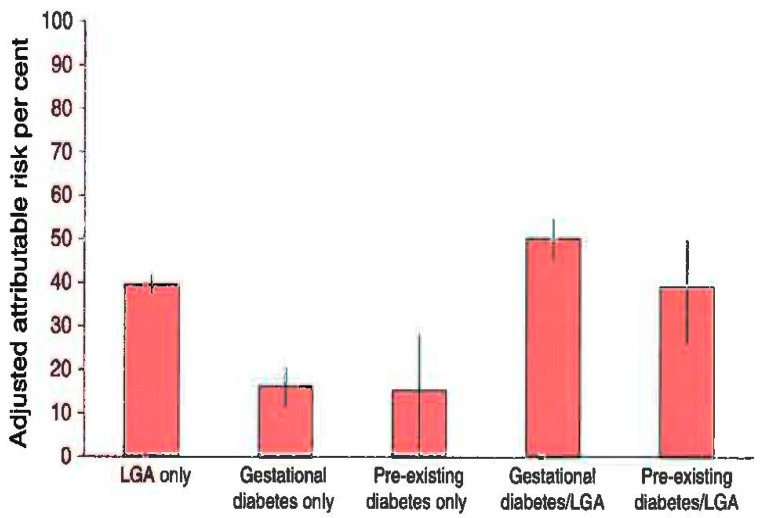
GDM, birth weight and risk for childhood obesity (Kaul et al., Diabetologia 2019) [82]. LGA = large for gestational age.

Maternal obesity and diabetes during gestation may heighten the risk of future cardiometabolic disorders in offspring. The study of Mitanchez D et al. in 2014 reports that the offspring of mothers with diabetes or obesity had a higher percentage of increased weight and obesity in adolescence (9.7% vs. 6.6% without GDM exposure) and a six-times-greater risk of T2D and insulin resistance [72]. The risk for T2D development was greater in the offspring of diabetic mothers (OR = 5.7) compared with the offspring of the obese group (OR = 2.8). Furthermore, the risk was 3.6 times greater for macrosomic babies compared with neonates with normal birth weight both in women with gestational diabetes and women with obesity. Finally, the combined fetal exposure in both diabetes and obesity during pregnancy was associated with an even greater risk (OR = 4.7) for future development of T2D in offspring. It is notable that 47.2% of young adults with T2D had a history of intrauterine exposure to GDM and obesity, as it is demonstrated in Figure 1.

According to Figure 1, the risk for childhood obesity was greater for the group of 4–6-year-old children who were born with macrosomia (40%). The children with a history of both maternal diabetes and neonatal macrosomia faced a greater risk of the future development of increased weight gain and obesity (50%) [82].

According to Hammoud et al., offspring born of mothers with T2D exhibited higher levels of BMI during adolescence, while intermediate BMI values were found in offspring of women with a history of gestational diabetes [83]. On the contrary, children whose mothers had T1D exhibited lower BMI values, possibly attributed to optimal diabetic control and a lower level of insulin resistance. Furthermore, higher levels of BMI were observed in adolescents who were born with macrosomia compared with those who had normal birth weight [83]. Thus, apart from genetic and epigenetic predisposition, environmental causes (including intrauterine environment) are also responsible for a higher risk in the development of childhood obesity.

The metabolic and vascular long-term GDM-related consequences also include a higher risk of pediatric and adolescent obesity, leading to precocious puberty and early growth spurts, hypertension, dyslipidemia, non-alcoholic fatty liver disease (NAFLD) and later in life the development of metabolic syndrome [35,84,85,86].

**F3. Obesity, type 2 diabetes, hypertension and offspring dyslipidemia**

The long-term cardiometabolic complications of GDM are described by the prospective Danish National Birth Cohort [87]. This study included 91,827 women between 1996 and 2002 from which 1350 had gestational diabetes and 2600 were the control group. Data was retrieved through telephone interviews. According to the results, adolescents with a history of maternal GDM exposure exhibited higher levels of BMI, waist/hip circumference, percentage of abdominal fat, systolic blood pressure, triglyceride levels, blood glucose, insulin levels, HOMA-IR and precocious puberty and lower HDL levels in comparison to unexposed adolescents. Consequently, adolescents that were exposed in utero to maternal diabetes are at greater risk of weight gain, obesity, T2D and later on possibly metabolic syndrome. Thus, hyperglycemia during pregnancy contributes to metabolic programming of offspring in adult life [87,88].

### 3.2. Gestational Obesity

#### 3.2.1. Gestational Obesity Demographics and Diagnosis

Gestational obesity is usually identified as an entity following the first appointment to the antenatal clinic. According to the American College of Obstetricians and Gynecologists (ACOG) and NHS UK, gestational obesity is the situation defined as BMI > 30 that poses great risks for the mother and the fetus/offspring [89,90]. Body Mass Index (BMI) is a simple and easy tool for identifying obesity. Nevertheless, it has certain limitations in distinguishing fat mass from muscle mass, and therefore women with increased muscle mass may be mistakenly taken for obese despite the fact that their fat mass may even be below average. In addition, fat accumulation plays an important role in the overall morbidity and health outcomes of these patients (abdominal obesity vs. thigh/hips obesity). Thus said, BMI is widely accepted as the main tool to identify obesity despite these limitations.

For the past few decades, there has been a rapid increase in the prevalence of obesity in both women in gestation as well as the general population. The global prevalence of maternal obesity is estimated to be 20.9% (95%CI 18.6–23.1%) and is expected to rise up to 23.3% (95%CI 20.3–26.2%) in 5 years [91]. That means that every year up to 39,000,000 women in gestation are identified as obese in the world, making maternal obesity considered an epidemic, bearing a heavy socioeconomic impact for many countries [92,93].

**A. GESTATIONAL-OBESITY-RELATED MATERNAL COMPLICATIONS**

Female obesity is considered an obstacle for women that wish to conceive, as it has been associated with low fertility rates, partially due to the coexistence of polycystic ovary syndrome (PCOS) [94]. Furthermore, women with gestational obesity experience higher rates of miscarriages, and therefore gestational obesity should be acknowledged as a medical condition that requires proper management when planning a pregnancy [94]. Measures such as healthy diet, weight reduction and exercise should be a major part of pregnancy planning in such cases.

Gestational obesity represents a major complication during pregnancy, implicated with significant short- and long-term morbidity for both the mother and offspring. As expected, pregnant women with obesity are at greater risk for gestational diabetes, hypertension, venous thromboembolism, preterm premature rupture of membranes, obstructive sleep apnea and pre-eclampsia (Table 5) [94,95,96,97].

**B. GESTATIONAL-OBESITY-RELATED OFFSPRING COMPLICATIONS**

Gestational obesity poses a great risk of serious complications for offspring, as it is associated with fetal macrosomia caused by subsequent alterations in fetal metabolism, shoulder dystocia, neural tube defects, stillbirth and others [95,96,97].

Gestational-obesity-related fetal macrosomia takes place via two mechanisms. The first mechanism involves insulin resistance and the second involves increased lipid availability and fetal lipid levels [68] (Table 5). As a consequence of macrosomia, shoulder dystocia may occur.

It is of great importance for the clinician overseeing a pregnancy complicated by GDM to understand that the risk of obesity-related congenital complications is weight-dependent, and therefore weight reduction is a major preventive measure. Nutrient deficiencies and metabolic disturbance caused by abnormal glucose levels have been associated with neutral tube defects, heart defects, cleft lip/palate, anorectal atresia, hydrocephaly and limb reduction anomalies [94]. Finally, as with pregnancies implicated with GDM, maternal obesity and excessive weight gain during pregnancy increase the risk for future obesity, T2D and cardiovascular disorders in offspring.

**C. LONG-TERM GESTATIONAL OBESITY OFFSPRING COMPLICATIONS**

#### 3.2.2. Diabesity-Related Complications

The pathophysiological mechanisms that explore the association of maternal obesity and diabetes with insulin resistance and fetal macrosomia are best described by Desoye G et al. [98]. Gestational diabetes and obesity can be regarded as two inseparable clinical entities, lately defined with the term “diabesity”. The offspring of mothers with diabesity exhibit increased fat deposition due to the excessive production and deposition of triglycerides under the state of fetal hyperinsulinemia. Insulin is a fascinating pancreatic hormone that exhibits, among other things, growth and mitogenic properties. Consequently, insulin can stimulate the production of white adipose tissue. Thus said, fetal insulin levels are highly dependent on fetal glucose levels and certain amino acid levels, such as arginine [99].

The placenta is responsible for the transport of nutrients to the developing fetus and therefore is of great significance for future fetal development. As such, the maternal environment in cases of gestational diabetes and obesity may lead to severe changes in placental development, contributing to fetal hyperglycemia at an early stage. Unfortunately, the fetal pancreas may be significantly affected by these changes with the acceleration of b-cell maturation and insulin hypersecretion. Fetal hyperinsulinemia is highly associated with the future development of obesity and T2D [100]. Thankfully, only a few cases of fetal hyperinsulinemia persist postnatally [100].

#### 3.2.3. Epigenetics in Diabesity

The term epigenetic alterations refers specifically to changes in DNA expression and not in DNA sequencing. Maternal diabesity triggers fetal epigenetic changes, such as macrosomia, and heightens the risk for future obesity and T2D [101]. Recently, Alba-Linares et al. observed DNA methylation during child development, especially in the first 6 months of life and up to the first year of life in infants born to mothers with obesity or obesity and gestational diabetes [101]. Enrichment analysis showed that DNA alterations affect genes and pathways associated with fatty acid metabolism, postnatal development and mitochondrial bioenergetic mechanisms (CPT1B, SLC38A4, SLC35F3 and FN3K). The maternal metabolic environment is very important for the developing fetus and has a significant impact in fetal programming, which in turn may result in future cardiovascular, metabolic and neurodevelopmental consequences [101].

Interestingly, the Lehnen et al. study underlined that maternal diabetes and obesity may have a negative impact on offspring wellbeing [102]. Furthermore, there seem to be notable differences among placental histone methylation in neonates of women with diabetes and obesity compared to healthy controls. The level of placental histone methylation is higher in neonates with no diabetes exposure, lower in neonates with maternal diabetes exclusively under diet modifications and lower in neonates with maternal diabetes under insulin therapy. Furthermore, the low level of methylation is negatively related to future obesity development. Thus said, the study of Dutton HP et al. showed that maternal obesity may negatively affect the mother (F0), the developing fetus (F1) and the embryonic germ cells (F2) and increase the risk for obesity for the mother and the subsequent two generations [94]. Therefore, therapeutic approaches should be initiated periconceptually or during pregnancy and to a lesser extent postnatally and through adulthood [103].

### 3.3. Preventional Measures of GDM and Obesity Complications

- Optimal glycemic control

Optimal glycemic control (HbA1c < 7%) is mandatory for cases of pregestational maternal diabetes with the objective of ensuring optimal fetal development and preventing the occurrence of congenital malformations. Furthermore, an oral glucose tolerance test with 75 mg glucose (OGTT) and fasting glucose measurements must be performed early during gestation. Recommendations for women with pregestational diabetes include adherence to a diabetes-specific dietary regimen, regular exercise, daily capillary glucose measurements, insulin treatment or antidiabetic medication in refractory cases (i.e., maternal MODY and PCOS), avoidance of excessive weight gain during pregnancy, antioxidant supplementation (folic acid, Mg, Fe, Ca and vitamin D in cases of deficiency) and regular ultrasonographic evaluation of the femoral/omphalic artery as per guidelines [104,105].

b. Weight management and healthy lifestyle

As mentioned above, gestational obesity furthermore possesses a great risk for maternal and fetal complications and, therefore, ACOG advises all women planning a pregnancy or in gestation to adopt a healthy lifestyle to minimize the possibility of an adverse outcome [5]. Overall lifestyle modifications can alter the modifiable predisposing GDM factors and improve overall maternal and fetal health and metabolic function. Therefore, among the strategies during pregnancy for the prevention of the reduction in fetal hyperinsulinemia are custom diet plans and enhanced physical activity even prior to conception [106]. Healthy lifestyle includes monitoring any excess weight gain before or during pregnancy, exercise and ensuring a healthy and stable enough diet to nurture the fetus and avoid LGA complications and malnourishment (never below 2000 kcal). A careful diet schedule and increased physical activity prior to conception may contribute to lowering the risk of fetal hyperinsulinemia [105].

c. Administration of Ω3 and folic acid

Maternal nutrition holds a key role in fetal growth and birth outcome. Exempli gratia, previously published study results indicate that adequate consumption of Ω3 fatty acids normalizes neurotrophin levels, reduces oxidative stress and contributes to the prevention of fetal neurological damage [107,108,109]. Finally, the supplementation with folic acid preconceptionally and until the closure of the neural tube (12 weeks) is indicated for all women in gestation in order to minimize the risk of neonatal neural tube defects [110].

d. Breastfeeding and prevention of infantile overfeeding

Finally, exclusive breastfeeding for at least 6 months has been found to reduce the future risk of offspring being overweight or obese and developing type 2 diabetes [111,112]. Offspring born to mothers with GDM should avoid gaining excess weight in childhood and adolescence to prevent chronic cardiometabolic diseases.

## 4. Discussion

This study was conducted in the form of a narrative review and summarized all the complications caused by obesity and GDM. Its originality lies in the simple and up-to-date narrative of GDM and obesity, emphasizing the newly acquired data and recommendations.

Research data indicate that GDM is responsible for a lengthy list of immediate and late complications for the mother and offspring and is furthermore a strong predictor of future GDM, T2D and cardiovascular disease [30,31]. Therefore, the increasing worldwide prevalence of GDM has important socioeconomic implications for mothers and their offspring, contributing to the augmentation of diabetes and obesity pandemics, and requires further preventive strategies to be established worldwide [22,23,24,25]. Thus said, more targeted studies are required to unfold the molecular mechanisms of GDM and establish further preventive strategies.

Undoubtedly, GDM has been held responsible over the years for a long list of serious maternal and offspring complications. We summarized and listed the most serious and common maternal complications that can be further subcategorized into gestational, labor, post-labor and late complications. The immediate complications include gestational stress, preeclampsia, hypertensive and circulation disorders, infections, premature delivery and maternal mortality, while the long-term complications include GDM in future pregnancies, T2D in the next 10 years, increased risk of cardiovascular disease compared to general population, etc. [30,31,32,33].

GDM-related neonatal complications include morbidity, prematurity, respiratory distress syndrome, cardiomyopathy, macrosomia, microsomia, perinatal asphyxia, polycythemia, hypoglycemia, hypocalcemia and hypomagnesemia [113]. Although the aforesaid results were obtained mainly from observational studies and caution is required when taking into account these results, they are nevertheless indicative of the risks involved in pregnancies complicated with GDM. Therefore, neonates born of mothers with GDM should be followed up in a Neonatal Intensive Care Unit (NICU) for the management of the above acute post-labor complications of GDM. Moreover, they should undergo a cardiologic evaluation for the diagnosis of cardiomyopathy, with subsequent follow-up assessment in the following months.

Long-term consequences of GDM in childhood/adolescence and adulthood include excess weight gain and obesity, precocious puberty and early growth spurts, which can consequently result in impaired glucose tolerance, arterial hypertension, dyslipidemia, non-alcoholic fatty liver disease (NAFLD), metabolic syndrome and neurodevelopmental disorders [35,42,84]. Moreover, the maternal diabetic intrauterine environment has been strongly associated with T2D development in offspring, and therefore these children should be eligible for future screening for diabetes and metabolic syndrome [33,114]. As the need for more efficient preventive measures emerges, future research is required to evaluate the necessity and frequency of future long-term follow-up of these patients including a 75gr OGTT and a cardiorespiratory evaluation. Moreover, during childhood/adolescence, offspring of GDM mothers should follow a hygiene diet and regular exercise in order to avoid obesity and its consequences.

As GDM predisposing factors include unmodifiable and modifiable elements, future preventive strategies against GDM should mainly and primarily focus on the research of predisposing modifiable factors. Among the aims of this study was to summarize all important preventive strategies against the development and management of GDM. As these factors may be reformed, GDM may be prevented with appropriate lifestyle modifications (weight loss and/or reduced weight gain during pregnancy, healthy diet and exercise) and medical treatment for cases of underlying endocrinological disorder such as PCOS [27].

As gestational obesity poses a great risk to both the mother and offspring, this study aimed to analyze the relationship between gestational obesity and adverse health outcomes for both the mother and offspring, which are similar to those of GDM, as well as to provide the most relevant research data in a simple and comprehensive way for the reader. Most research results indicate a strong positive relationship between gestational obesity and adverse health outcomes, such as macrosomia and caesarian delivery [92]. Therefore, gestational obesity is an important complication of pregnancy that needs to be addressed with great caution by clinicians.

Gestational diabetes and obesity are two inseparable clinical entities, now defined as “diabesity”, that heighten the risk of future cardiometabolic consequences in offspring. Thankfully, lifestyle modifications and exercise may minimize the risk of diabesity-related fetal hyperinsulinemia. Therefore, among the strategies before conception and during pregnancy for the prevention of the reduction in fetal hyperinsulinemia are custom diet plans and enhanced physical activity that should begin even during pregnancy planning [106].

GDM-related complications primarily involve a disturbed interplacental environment generated by an imbalance between pro-oxidative and antioxidant cellular systems [48]. Therefore, antioxidant pathways work as a salvation mechanism, mitigating oxidative damage caused by various maternal conditions. As such, research should further focus on internal and external protective antioxidant hydrophilic and hydrophobic substances [53,54,55,56,57]. Thus said, it has been widely accepted that multiple antioxidant substances may contribute to oxidative stress reduction during pregnancy. Vitamins C and E, lipoic acid and myoinositol may prevent fetal anomalies and placental vessel damage. Flavonoids like quercetin-coenzyme Q (a flavonoid which can be found in many fruits, vegetables and seeds) and resveratrol (a polyphenol found in foods like red grapes, blueberries, raspberries, peanuts and red wine) have powerful antioxidant, antiviral and cardioprotective actions through the improvement of lipid profile and LDL-C levels, as well as their beneficial effect on placental vessels [115,116]. Similarly to the supplementation of linoleic acid, myoinositol and vitamin E, which are highly recommended for the prevention of neural tube defects [117,118], vitamin C, lipoic acid, flavonoids and resveratrol ought to be further investigated in order to further prove their efficacy [115,116].

Folic acid, alertly known as vitamin B9, is a very important vitamin of the B complex that cannot be synthesized by the human body. As such, sufficient folic acid consumption must be provided through diet or dietary supplements. Folic acid is essential for cell proliferation through DNA and RNA synthesis, the transfer of genetic information and cell division in the first trimester of pregnancy. Moreover, it is essential for red blood cell production, neural tissue development and other human body tissues. Folic acid supplementation during pregnancy enhances levels of antioxidant enzymes and reduces levels of apoptotic proteins and lipid peroxidation, thus preventing neurodevelopmental disorders associated with GDM [119].

In addition, polyunsaturated long-chain fatty acids have been studied as antioxidant agents that could potentially diminish the production of ROS and NOS, thereby contributing to the prevention of congenital defects of the fetal central nervous system. The supplementation of an “antioxidant agents mix” containing linoleic acid, myoinositol and vitamin E is highly recommended during pregnancy for neural tube defect prevention in offspring of mothers with pregestational diabetes. This cocktail must be given 3 months before conception but also during the first trimester of pregnancy, as it has been proven that it may hinder the development of neural tube defects [117,118].

Finally, the prevention of excessive weight gain during the first months of life, especially through breastfeeding, in infants born to mothers with GDM and/or obesity has been found to lower any future risk related to obesity and T2D in these children [111,112]. Therefore, breastfeeding campaigns should further highlight the importance of breastfeeding against intergenerational complications. Consequently, pediatricians should recommend that mothers not only extend exclusive breastfeeding for at least 6 months but also avoid overfeeding, known as infantile excess feeding, during the first year of life, in order to prevent obesity and the future development of T2DM and metabolic syndrome.

## 5. Conclusions

Gestational hyperglycemia leads to oxidative and nitrogen stress development and synchronous inhibition of antioxidant mechanisms, resulting in severe maternal, fetal and placental morbidity. Diabetes-related interplacental functional alterations to fetal programming against oxidative stress and fetal macrosomia result in complications such as septal hypertrophy, neurodevelopmental disorders, obesity, precocious puberty, type 2 diabetes and metabolic syndrome later in life. Moreover, neonatal macrosomia is associated with many immediate neonatal adverse events as well as long-term complications for offspring.

Maternal obesity may have negative impacts similar to maternal diabetes, as it causes fetal macrosomia and cardiometabolic complications later in life. Optimized glycemic control may be an excellent preventing agent against oxidative stress in diabetic pregnancies. In that view, pregnant women should also receive folic acid, regular exercise and weight monitoring during pregnancy.

Breastfeeding has a positive impact on infants born to mothers with diabesity. Again, weight monitoring during the first months may be crucial in avoiding the development of obesity and metabolic syndrome later in life.

## 6. Future Directions

GDM has emerged as a significant global health matter, posing serious risks for both mothers and offspring along with serious intergenerational health complications. As such, it is important to effectively address GDM with caution and in a multidisciplinary approach.

First, as fetal reprogramming and oxidative stress mechanisms have been accused of causing serious adverse outcomes regarding fetal health, we propose shifting the focus towards the GDM-related oxidative stress consequences, which are responsible for a broad range of interplacental complications affecting both the mother and the fetus. Therefore, further research should be conducted on the use of antioxidant supplementation during pregnancy as well as a preconception measure for mothers with PDGM and previous history of GDM while planning for their future pregnancy.

In that view, additional studies are needed that emphasize the use of continuous glucose monitoring (CGM) systems and adherence to stricter specific GDM guidelines and dietary modifications during pregnancy, as tight glucose monitoring and optimal surveillance have been shown to be essential components of effective GDM treatment [120]. Moreover, the use of HbA1c should be further studied, as HbA1C, although unreliable in pregnancy, is a simple and cost-effective tool in diabetes surveillance. Although recent studies indicate that HbA1c is not as sensitive and specific as OGTT in the first trimester, further research may be needed in order to re-evaluate the role of HbA1c in pregnancy [121].

Although there is no doubt that scientific research and progress have been made towards the treatment of GDM, there is still an uncovered area of knowledge in therapeutic alternatives to insulin such as lifestyle modification, metformin and glyburide and their long-term effectiveness.

Concerning preventive strategies and health measures against GDM and GDM-related complications, future research should primarily redirect the focus on the long-term effect of regular physical activity on oxidative stress and antioxidant mechanisms in women with diabetes during gestation (either GDM or PDGM). Additionally, studies should investigate the impact of regular mild–moderate exercise against oxidative stress during gestation and as a preconception modification and protective mechanism against complications.

As obesity poses a great threat to overall health, new strategies against it should be proposed. As such, further research should be conducted in mothers with obesity during gestation, as obesity may be responsible for fetal reprogramming, implicated with later cardiometabolic complications.

Finally, there should be further research into the late complications of diabesity in future generations, as fetal reprogramming and free radicals may be responsible for the intergenerational late sequelae of diabesity during gestation.

## Figures and Tables

**Table 1:** Prisma Flow diagram.

**A.** GDM epidemiology and oxidative stress.
**B.** Obesity in pregnancy and effects on the mother.
**C.** GDM prevention complications for the mother and offspring.
** 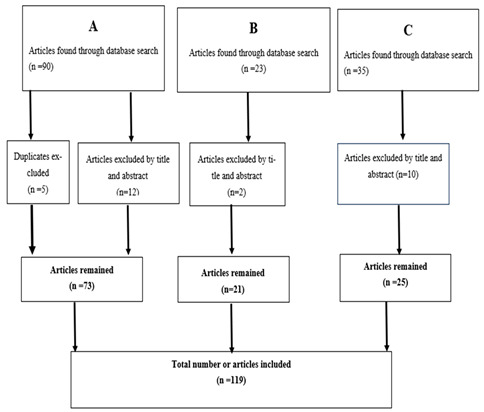 **

**Table 2:** Gestational diabetes diagnosis [26].

**During the First Visit of the Pregnant Woman**
▪ If fasting glucose > 126 mg/dL -> pre-existing diabetes ▪ If fasting glucose > 92 mg/dL but <126 mg/dL -> gestational diabetes ▪ If fasting glucose < 92 mg/dL -> glucose–insulin curve at 24–28 gestational weeks for all pregnant women
**Glucose–Insulin Curve (with Administration of 75 g Glucose)**
▪ Serum fasting glucose > 92 mg/dL * ▪ Serum glucose 1st hour > 180 mg/dL * ▪ Serum glucose 2nd hour > 153 mg/dL *

(* diagnostic of GDM).

**Table 4:** Consequences of uncontrolled pregestational maternal diabetes on the fetus [40,42,46,47].

Affected System	Congenital Malformations
Congenital heart diseases	Truncus arteriosus Ventricular septal defect Transposition of great arteries Tricuspid atresia Single ventricle complex
Musculoskeletal anomalies	Sacral agenesis Meningocele Longitudinal limb deficiencies
Other congenital malformations	Anal agenesis Hypoplastic kidneys Renal agenesis Cleft palate
Central nervous system defects	Holoprosencephaly Anencephaly Hydrocephalus Anotia/microtia Craniorachischisis
Neurodevelopmental disorders	Attention deficit-hyperactivity disorder (ADHD) Autism Fine and gross motor disorders Psychiatric diseases (schizophrenia and anxiety disorder)

**Table 5:** Gestational obesity related to immediate maternal and fetal complications [94,95,96,97].

Maternal complications	Hypertension Postpartum hemorrhage
	GDM
	Preeclampsia
	Obstructive sleep apnea Preterm premature rupture of membranes Postpartum venous thromboembolism Labor induction Caesarian section
Fetal/neonatal complications	Macrosomia Shoulder dystocia
	Large and small for gestational age (SGA and LGA) Neural tube defects and cerebral palsy Hydrocephaly Anorectal atresia Cardiac defects Cleft lip/palate
	Stillbirth

## Data Availability

The original contributions presented in this study are included in the article. Further inquiries can be directed to the corresponding author.
